# 5AtRabD2b and AtRabD2c have overlapping functions in pollen development and pollen tube growth

**DOI:** 10.1186/1471-2229-11-25

**Published:** 2011-01-26

**Authors:** Jianling Peng, Hilal Ilarslan, Eve Syrkin Wurtele, Diane C Bassham

**Affiliations:** 1 Department of Genetics, Development and Cell Biology, Iowa State University, Ames, IA 50010, USA

## Abstract

**Background:**

Rab GTPases are important regulators of endomembrane trafficking, regulating exocytosis, endocytosis and membrane recycling. Many Rab-like proteins exist in plants, but only a subset have been functionally characterized.

**Results:**

Here we report that AtRabD2b and AtRabD2c play important roles in pollen development, germination and tube elongation. *AtrabD2b *and *AtrabD2c *single mutants have no obvious morphological changes compared with wild-type plants across a variety of growth conditions. An *AtrabD2b/2c *double mutant is also indistinguishable from wild-type plants during vegetative growth; however its siliques are shorter than those in wild-type plants. Compared with wild-type plants, *AtrabD2b/2c *mutants produce deformed pollen with swollen and branched pollen tube tips. The shorter siliques in the *AtrabD2b/2c *double mutant were found to be primarily due to the pollen defects. *AtRabD2b *and *AtRabD2c *have different but overlapping expression patterns, and they are both highly expressed in pollen. Both AtRabD2b and AtRabD2c protein localize to Golgi bodies.

**Conclusions:**

These findings support a partially redundant role for AtRabD2b and AtRabD2c in vesicle trafficking during pollen tube growth that cannot be fulfilled by the remaining AtRabD family members.

## Background

Ras-like small GTP-binding proteins (GTPases) regulate diverse processes in eukaryotic cells including signal transduction, cell proliferation, cytoskeletal organization and intracellular membrane trafficking. GTPases are activated by GTP binding and inactivated by subsequent hydrolysis of bound GTP to GDP, thus acting as molecular switches in these processes [1,2]. The Rab GTPase family is the largest and most complex within the Ras protein superfamily. Rab GTPases are important regulators of endomembrane trafficking, regulating exocytosis, endocytosis and membrane recycling processes in eukaryotic cells [3-6]. Rab GTPase functions have been extensively studied in yeast and mammalian systems. Both *in vivo *and *in vitro *experiments have demonstrated that different Rab proteins function in distinct intracellular membrane trafficking steps and they are hypothesized to work together with soluble *N*-ethylmaleimide-sensitive factor attachment protein receptor (SNARE) proteins to promote specificity of vesicle transport to target compartments and facilitate vesicle and target membrane fusion [7-13]. They are therefore essential for the transport of proteins and membrane through the endomembrane system to their destination.

The *Arabidopsis thaliana *genome encodes 93 putative Ras superfamily proteins. Fifty-seven of these are Rab GTPases, more than in yeast but similar to the number in humans [13,14]. According to their sequence similarity and phylogenetic clustering with yeast and mammalian orthologs, these Rab proteins were assigned to eight subfamilies, AtRabA to AtRabH, which can be further divided into 18 subclasses [13]. Relatively few of the plant Rab orthologs have been investigated functionally. Most of these studies have used constitutively active (CA) and/or dominant negative (DN) mutations, generated by direct mutation of the conserved domain to restrict mutant GTPase proteins to the active GTP-bound form (constitutively active) or inactive GDP-bound form (dominant negative). Expression of CA or DN Rab GTPases can perturb the activity of the endogenous Rab, revealing their functional significance. For a number of plant Rab GTPases, expression of their CA and DN mutants in transformed plants, together with protein localization information, has shown that these Rabs perform functions similar to those of their yeast and mammalian orthologs [15-19].

Several reports indicate that Rab proteins are important for elongation of tip-growing cells in plants. For example, AtRabA4b is reported to localize to the tips of root hair cells and was proposed to regulate membrane trafficking through a compartment involved in the polarized secretion of cell wall components [18]. NtRab2 GTPase is important for trafficking between the endoplasmic reticulum and Golgi bodies in tobacco pollen tubes and may be specialized to optimally support the high secretory demands in these tip growing cells [16]. NtRabA (Rab11) in tobacco is predominantly localized to an inverted cone-shaped region at the pollen tube tip, and both constitutively active and dominant negative mutants resulted in reduced tube growth rate, meandering pollen tubes, and reduced male fertility [20].

There are four genes in the Arabidopsis RabD subfamily, *AtRabD1 *(At3g11730), *AtRabD2a *(At1g02130, AtRab1b), *AtRabD2b *(At5g47200, AtRab1a) and *AtRabD2c *(At4g17530, AtRab1c) [13]. In mammals, the orthologs of AtRabD, Rab1 isoforms, physically associate with the ER, ER-Golgi intermediate compartment and Golgi and regulate membrane trafficking between the ER and Golgi complex [21]. Fluorescent protein fusions with AtRabD1, AtRabD2a and AtRabD2b localize to the Golgi and *trans*-Golgi network [22,23], and transient expression in plant cells of dominant negative mutants of *rabD2a *or *rabD1 *resulted in the inhibition of ER-to-Golgi trafficking [15,22,24], suggesting a related function for the plant Rab1 homologs. Pinheiro et al. [22] isolated T-DNA insertion mutants in each of the AtRabD family genes and reported that each of the single and double mutants lacked a detectable phenotype. By contrast, a *rabD2a rabD2b rabD2c *triple mutant was lethal and a *rabD1 rabD2b rabD2c *triple mutant had stunted growth and low fertility, indicating that these gene family members perform important and overlapping functions.

We previously hypothesized that closely related genes with a high Pearson correlation in their RNA accumulation level are functionally redundant, and showed that expression patterns of both the *AtRabD2b *and *AtRabD2c *genes are negatively correlated with the process of starch synthesis [25], whereas the expression patterns of the remaining *RabD *genes are not. We therefore predicted that these two Rab proteins may have redundant functions that are not shared by the other two AtRabD family members. Here we show that AtRabD2b and AtRabD2c are highly correlated in their RNA accumulation level across a variety of experimental conditions. Phenotypic analysis of knockout mutants indicates that they are at least partially functionally redundant, and are important in pollen development and pollen tube growth. The proteins both localize to the *trans*-Golgi, consistent with their proposed role in trafficking from the ER to the Golgi apparatus.

## Results

### The expression patterns of *AtRabD2b *and *AtRabD2c *are closely correlated

The four RabD family members in Arabidopsis share about 88% identity at the amino acid level. The accumulation pattern of the associated transcripts is quite distinct across a wide variety of experimental conditions and developmental stages (MetaOmGraph, http://www.metnetdb.org/MetNet_MetaOmGraph.htm;[26]) (Table 1; Additional file 1, Table S1). *AtRabD2b *and *AtRabD2c *expression patterns are correlated (at a Pearson correlation value of 0.72), whereas *AtRabD1 *and *AtRabD2a *show very low correlation with the others (Pearson correlation value of < 0.20). Based on their high sequence similarity (99% amino acid identity) and the correlation between their mRNA accumulation patterns, we hypothesized that AtRabD2b and AtRabD2c might have some functional overlap that is not shared by AtRabD1 and AtRabD2a.

**Table 1:** Pearson correlation between expression patterns of AtRabD family members.

	*AtRabD1*	*AtRabD2a*	*AtRabD2b*	*AtRabD2c*
***AtRabD1***	100%			

***AtRabD2a***	17.08%	100%		

***AtRabD2b***	-3.4%	22.19%	100%	

***AtRabD2c***	15.92%	25.23%	**77.81%**	100%

### Identification of Null Mutations in the Genes *AtRabD2b *and *AtRabD2c*

**Figure 1:**
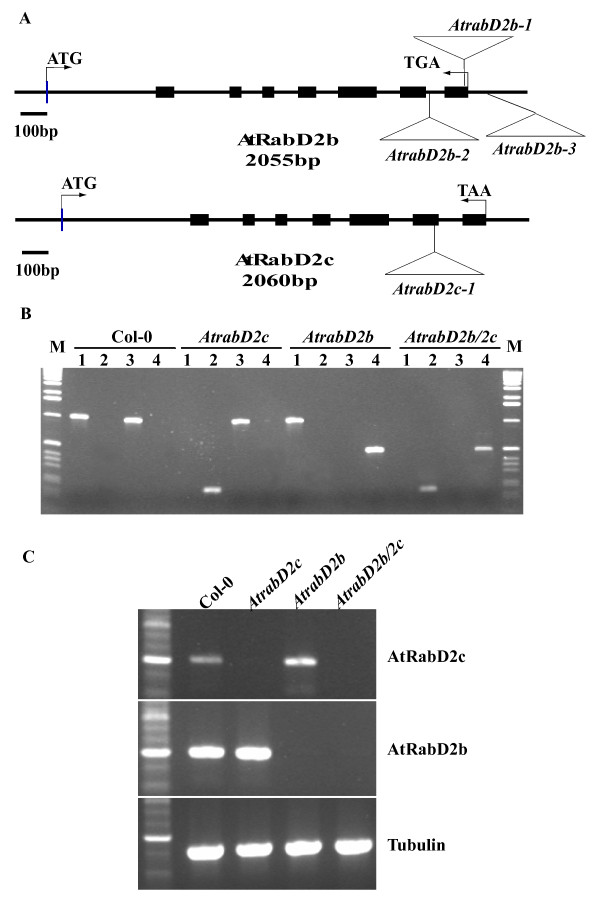
**Characterization of *AtrabD2b *and *AtrabD2c *mutations**. A, Gene map. The scaled linear map depicts the 8 exons as boxes and the 7 introns as lines between the boxes for both the *AtRabD2b *and *AtRabD2c *genes. The positions of the translational start and stop codons in exon 1 and exon 8, respectively, are noted. The locations of the T-DNA insertions (not drawn to scale) in the genes are indicated. B, Genotypes of T-DNA insertion mutants. Genomic DNA was isolated from the indicated single and double mutants and amplified by PCR. Primer pairs used were as following: lane 1, *AtrabD2c*-LP1 and *AtrabD2c-*RP1; lane 2, *AtrabD2c-*RP1 and LBb1; lane 3, *AtrabD2b*-LP1 and *AtrabD2b*-RP1; lane 4, *AtrabD2b*-RP1 and LBb1. C, Analysis of transcripts from *AtrabD2b-1*, *AtrabD2c-1 *and *AtrabD2b/2c *mutants. Total RNA from leaves of wild-type plants, *AtrabD2c-1*, *AtrabD2b-1 *and *AtrabD2b/2c *was amplified by RT-PCR. Primer pairs for *AtRabD2c *were AtRabD2c-F and AtRabD2c-R, primer pairs for *AtRabD2b *were AtRabD2b-F and AtRabD2b-R. Tubulin was used as control.

It was reported previously that an *AtrabD2b AtrabD2c *double mutant has no phenotype [22]. Based on our correlation analysis above, we hypothesized that this mutant may have some more subtle defects that cannot be compensated for by the remaining family members. To investigate this further, we identified T-DNA insertion mutants (Figure 1A) in *AtrabD2b *(3 alleles) and *AtrabD2c *(1 allele). Homozygous lines for the T-DNA insertions were identified by PCR, using primers selected by iSct primers (http://signal.salk.edu/tdnaprimers.2.html), and the insertion sites were determined by sequencing the PCR products (Figure 1B). Analysis of mRNA levels by RT-PCR indicated that *AtrabD2b-1*, *AtrabD2b-2 *and *AtrabD2c-1 *are null mutants. However, the *AtrabD2b-3 *mutation had no effect on *AtRabD2b *RNA accumulation (Figure 1C and data not shown). Progeny from *AtrabD2b-1 *and *AtrabD2c-1 *heterozygotes showed a T-DNA segregation ratio of approximately 3:1 based on kanamycin resistance, consistent with a single insertion. *AtrabD2b-2 *was supplied as a homozygous line. To generate *AtrabD2b AtrabD2c *double mutants, *AtrabD2b-2 *and *AtrabD2c-1 *homozygous single mutants were crossed, F1 plants were allowed to self fertilize and the *AtrabD2b-2/AtrabD2c-1 *double mutant was identified from the F2 population by PCR using the primers for both *AtrabD2b-2 *and *AtrabD2c-1*. Hereafter, the *AtrabD2b-2/AtrabD2c-1 *double mutant will be referred to as *AtrabD2b/2c*, and *AtrabD2b-2 *and *AtrabD2c-1 *single mutants will be referred to as *AtrabD2b *and *AtrabD2c *respectively.

### Siliques Are Shorter in the *AtrabD2b/2c *Double Mutant than in Either Single Mutant or in Wild-Type Lines

To evaluate phenotypes associated with the *AtrabD2b *and *AtrabD2c *mutants, homozygous *AtrabD2b *(three alleles, *AtrabD2b-1*, *AtrabD2b-2 *and *AtrabD2b-3*), *AtrabD2c *and *AtrabD2b/2c *mutants, along with wild-type siblings, were grown on agar plates with or without various hormone, nutrient and light treatments. We tested over 50 of the conditions described in the Gantlet website (http://www.gantlet.org); however, no significant phenotypic differences were observed in the seedlings for any of the mutant alleles (data not shown). In addition, we tested the seedling phenotype on media with or without sucrose or vitamin B5 and, consistent with previous reports [22], no obvious phenotypes were observed.

**Figure 2:**
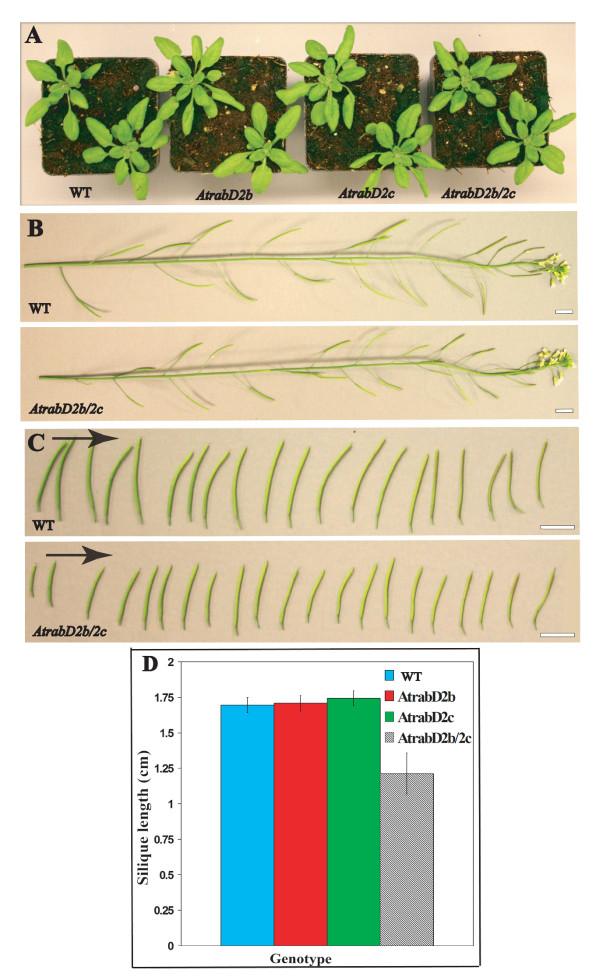
**The *AtrabD2b/2c *double mutant shows a striking shorter silique phenotype**. A, Vegetative growth of *AtrabD2b*, *AtrabD2c *and *AtrabD2b/2c *plants. B, Inflorescence of *AtrabD2b/2c *and wild-type plants. Scale bars = 850 μm. C, Siliques from the *AtrabD2b/2c *mutant and wild-type plants; arrows indicate the sequence of siliques from the oldest to the youngest. Scale bars = 850 μm. D, Siliques (from 6 to 14 ) of the first inflorescence for wild type, single and double mutants were measured for each plant, with 10 plants measured for each genotype. Error bars indicate standard deviation.

**Figure 3:**
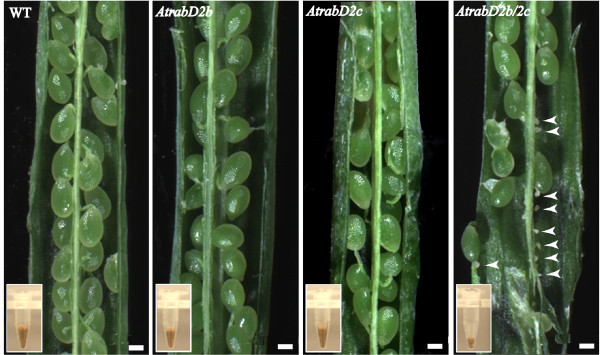
**There are many non-fertilized ovaries in the *AtrabD2b/2c *double mutant**. Individual siliques of wild type, single and double mutant plants were dissected and examined under the microscope. Arrows heads indicate unfertilized ovaries. Inset, seeds produced by a single plant. Scale bars = 200 μm.

By contrast, *AtrabD2b/2c *double mutant lines showed a phenotype associated with reproduction. In these lines, siliques were shorter when grown either under continuous light or long day (16h light/8h dark) conditions. Neither the *AtrabD2b *nor the *AtrabD2c *single mutant alleles displayed a short silique phenotype. The length of *AtrabD2b/2c *siliques was 70% of that of wild-type, *AtrabD2b *or *AtrabD2c *single mutant lines (Figure 2; P < 0.01 by Student's *t*-test). To evaluate whether this reduced silique size is associated with a seed defect, siliques from *AtrabD2b/2c*, wild-type, *AtrabD2b *and *AtrabD2c *mutant lines were opened at 10 DAF (days after flowering). Consistently, no defects in the seeds of either *AtrabD2b *or *AtrabD2c *single mutants were observed. However, approximately half of the ovules in the *AtrabD2b/2c *double mutant were not fertilized (Figure 3). Consistent with this observation, the *AtrabD2b/2c *mutant plants produced a smaller quantity of seeds than wild-type plants or single mutants (Figure 3; Additional file 2, Figure S1). These results are consistent with a functional overlap between AtRabD2b and AtRabD2c that cannot be fulfilled by AtRabD1 or AtRabD2a.

### Complementation of *AtrabD2b/2c *Mutant Phenotype

**Figure 4:**
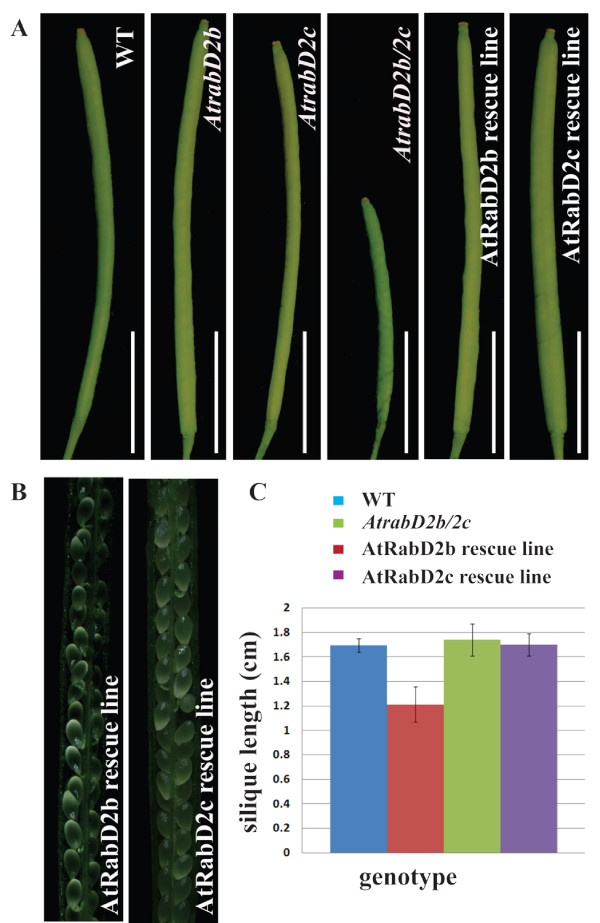
**Complementation of the double mutant phenotype**. A, Siliques are shown from wild-type plants, *AtrabD2b *and *AtrabD2c *single mutants, the *AtrabD2b/2c *double mutant and the *AtrabD2b/2c *double mutant complemented with either *AtRabD2b *or *AtRabD2c*. Scale bars = 0.5 cm. B, Individual siliques of rescued lines were dissected and examined under the microscope. Scale bars = 600 μm. C, Siliques (from 6 to 14 ) of the first inflorescence for the indicated genotypes were measured for each plant, with 10 plants measured for each genotype. Error bars indicate standard deviation.

To demonstrate that the *AtrabD2b/2c *mutant phenotype is due to the mutations in the *AtRabD2b *and *AtRabD2c *genes, constructs containing either *AtRabD2b *or *AtRabD2c*, each expressed from their native promoter, were introduced into the *AtrabD2b/2c *double mutant. Both constructs were able to rescue the silique length phenotype of the mutant (Figure 4A, C) and restored the seed fertilization defect (Figure 4B) and seed number (Additional file 2, Figure S1), confirming that the loss of AtRabD2b and AtRabD2c is responsible for these phenotypes.

### *AtrabD2b/2c*, *AtrabD2b *and *AtrabD2c *Pollen Have Defects in Morphology and Pollen Tube Elongation

**Figure 5:**
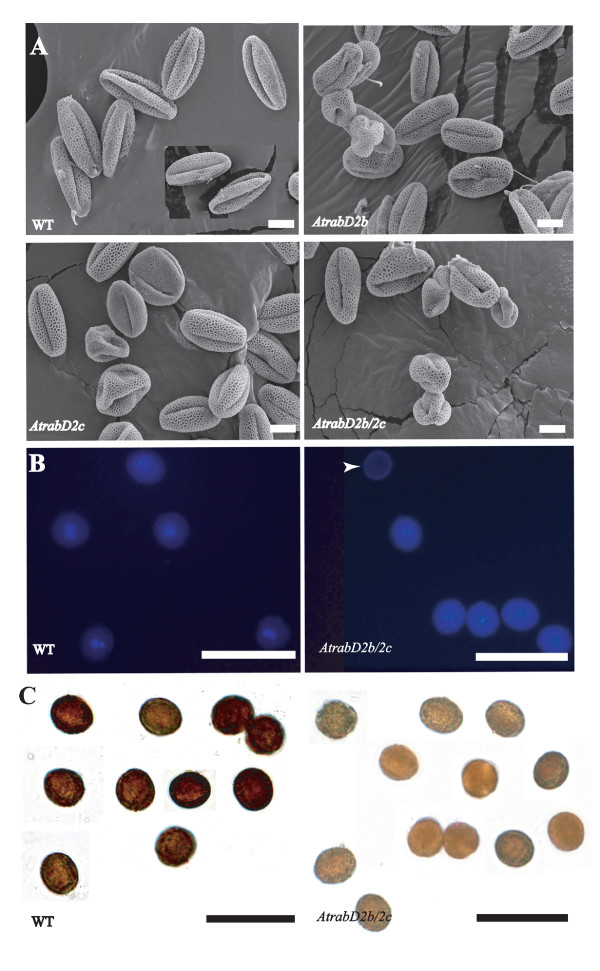
**Pollen defects in *AtrabD2b*, *AtrabD2c *and *AtrabD2b/D2c *mutants**. A, Fresh pollen was examined by SEM. B, DAPI staining of pollen. Fresh pollen grains were stained with DAPI and photographed under the fluorescence microscope. Arrow indicates a pollen grain from the *AtrabD2b/2c *mutant that lacks a nucleus. C, IKI staining of pollen, demonstrating reduced staining of the *AtrabD2b/2c *double mutant pollen compared with wild-type pollen. Scale bars = 10 μm (A); 50 μm (B,C).

Two possibilities could explain the unfertilized embryos seen in the *AtrabD2b/2c *double mutants. One possibility is that the pollen bears a defect that leads to pollen sterility and inability to fertilize the embryos. Alternatively, ovules may bear an abnormality such that their fertilization is reduced. To distinguish between these two possibilities, we observed the pollen by scanning electron microscopy (SEM). All of the pollen from wild-type plants looked normal, whereas more than 20% of the *AtrabD2b/2c *pollen exhibited an irregular, collapsed morphology (Figure 5A). We also observed that some abnormal pollen grains from the *AtrabD2b/2c *double mutant were devoid of nuclei, as indicated by DAPI staining, whereas all pollen from wild-type (Figure 5B) and single mutant plants (data not shown) have nuclei. This defective pollen may be the severely collapsed pollen visualized under the SEM. Surprisingly, even the *AtrabD2b *and *AtrabD2c *single mutant lines produce aberrant pollen at a level of about 10%. This is unexpected, as the *AtrabD2b *and *AtrabD2c *single mutants have normal-appearing siliques and seed quantities similar to the wild-type plants. A likely explanation is that there are sufficient normal pollen grains in the single mutants to efficiently fertilize the ovaries in the *AtrabD2b *and *AtrabD2c *single mutants.

We originally identified AtRabD2b and AtRabD2c because the transcript accumulation patterns of these two genes correlate with those of many genes associated with starch metabolism. Indeed, the *AtrabD2b/2c *double mutant pollen stained less intensely with IKI than wild-type pollen (Figure 5C), suggesting a decreased starch content in the *AtrabD2b/2c *mutant pollen. This is consistent with the expression correlation, although the reason for this phenotype is unclear.

**Figure 6:**
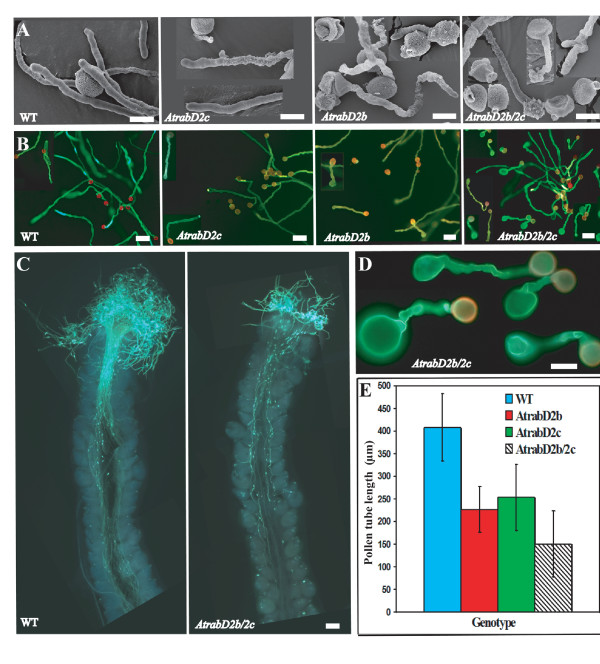
**Pollen tube elongation defects in *AtrabD2b*, *AtrabD2c *and *AtrabD2b/2c *mutants**. A, Pollen was germinated *in vitro *for 6 hours and examined by SEM. B, Germinated pollen was stained with aniline blue then observed under an epifluorescence microscope. C. Open flowers from an *AtrabD2b/2c *mutant plant, along with a wild-type plant, were incubated overnight on medium then examined by fluorescence microscopy. D. Close up view of pollen tubes in the *AtrabD2b/2c *mutant. E. Pollen was germinated *in vitro *and pollen tube length measured after an overnight incubation using SIS Pro software (OSIS, Lakewood, CO) (n > 200). Error bars indicate standard deviation. Scale bars = 10 μm (A); 50 μm (B, C); 20 μm (D).

A single flower of Arabidopsis produces thousands of pollen grains, but usually there are less than 100 embryos in one silique. If only 20% of the pollen grains are abnormal, we would not expect the strikingly reduced fertility seen in the *AtrabD2b/2c *double mutant. We therefore looked for additional explanations for the reduced fertility. To evaluate germination and tube growth of the pollen grains, pollen was germinated *in vitro*. After overnight incubation, almost all of the pollen from wild-type plants germinated and showed a typical tip growth. However, about 10% of the pollen from the *AtrabD2b/2c *mutant did not germinate at all and 50% of the pollen germinated but did not grow apically as did pollen of wild-type plants (Figure 6A and 6B). Instead, these pollen tubes were shorter and had swollen tips, some burst (≈5%), and others branched (≈2%; Figure 6A,B and 6D). The germination rate of the pollen from the single mutants was similar to the wild-type pollen. However, approximately 20% of the germinating pollen also had swollen tips (Figure 6A and 6B), although the phenotype was not as severe as the *AtrabD2b/2c *double mutant; burst or branched tubes were never observed in either single mutant (Figure 6A and 6B). Moreover, the pollen tubes of the *AtrabD2b/2c *double mutant were much shorter than those of wild-type plants or either single mutant (P < 0.01), and the single mutants had shorter pollen tubes than wild-type plants (Figure 6E; P < 0.01 for both mutants). Even though the *AtrabD2b *and *AtrabD2c *single mutants had collapsed pollen, shorter pollen tubes and swollen tips, their siliques were normal compared with wild-type plants. We hypothesize that the single mutants may still have sufficient normal pollen to enable all embryos to be fertilized. The *in vitro *pollen germination phenotypes were confirmed by analyzing pollen tube growth after *in vivo *pollination (Figure 6C). Open flowers from wild-type or *AtrabD2b/2c *mutant plants were incubated overnight on agar medium. The *AtrabD2b/2c *mutant flowers had reduced pollen germination and decreased pollen tube length compared with wild-type plants, suggesting that pollen germination and pollen tube growth may also be defective *in vivo*.

### Pollen and Pollen Tube Defects Cause the Shorter Siliques in the *AtrabD2b/2c *Mutant

**Figure 7:**
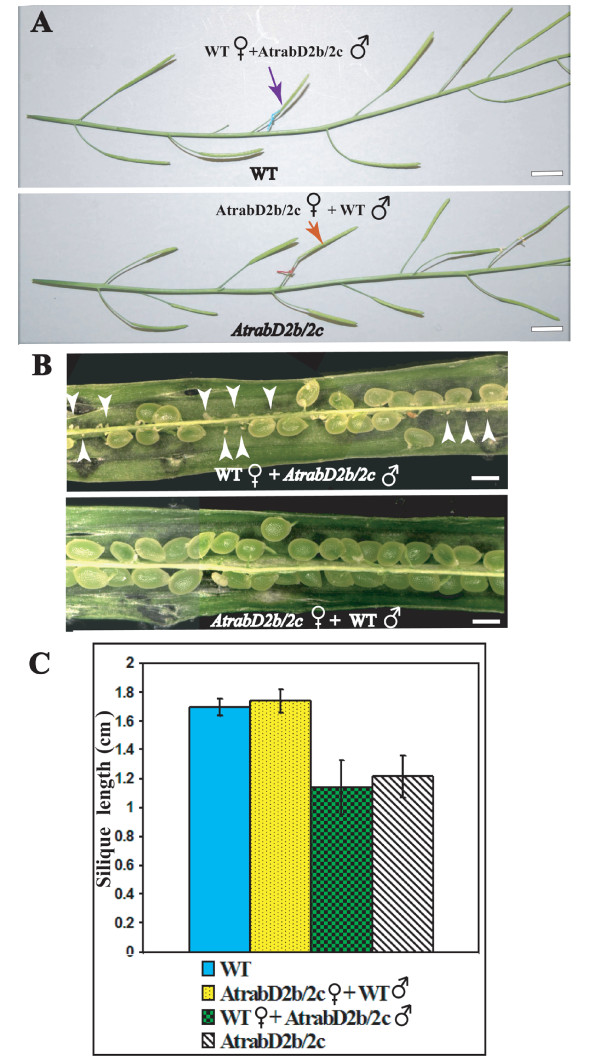
**Wild-type pollen can restore the shorter siliques of the *AtrabD2b/2c *mutant to normal length**. Wild-type and *AtrabD2b/2c *double mutant plants were crossed and silique length measured after 10 days. A. Inflorescences from a cross between a wild-type plant and *AtrabD2b/2c *mutant. The blue arrow indicates a silique in which a wild-type pistil was fertilized with *AtrabD2b;AtrabD2c *pollen. The red arrow indicates a silique in which the *AtrabD2b/2c *mutant pistil was fertilized with wild-type pollen. B. Siliques from the crosses at 10 DAP (days after pollination) were dissected and examined under a stereo microscope. White arrowheads indicate unfertilized embryos found upon pollination of wild-type plants with *AtrabD2b/2c *pollen. C, More than 20 siliques were measured for each plant. Error bars indicate standard deviation. Scale bars= 850 μm (A); 500 μm (B).

To investigate whether the unfertilized seeds are due to the observed pollen abnormality, or whether the ovary also has defects that might contribute to the reduced rate of fertilization, we crossed wild-type and *AtrabD2b/2c *mutant plants. If the shorter silique phenotype is borne only by the abnormal pollen, wild-type plant pollen should rescue the *AtrabD2b/2c *mutant silique phenotype to a normal length (*AtrabD2b/2c *mutant female flower crossed with wild-type plant pollen). In contrast, the *AtrabD2b/2c *mutant plant pollen crossed with a wild-type female would mimic the mutant phenotype of decreased fertilization (wild-type female flower crossed with *AtrabD2b/2c *mutant pollen). Alternatively, if the ovary also has some abnormality, wild-type pollen would not completely rescue the mutant phenotype, and *AtrabD2b/2c *mutant pollen would not mimic the mutant phenotype. The results of these crosses indicated that pollen from wild-type plants can rescue the *AtrabD2b/2c *short silique phenotype, and the pollen from *AtrabD2b/2c *can bestow the shorter silique phenotype on wild-type plants (Figures 7A and 7C). Specifically, about half of the seeds were not fertilized in the siliques that developed from wild-type pistils fertilized by *AtrabD2b/2c *pollen (Figure 7B). In contrast, the siliques from *AtrabD2b/2c *usually had about 50% unfertilized ovules, but when these pistils were fertilized by wild-type pollen, all seeds looked normal, and the siliques were longer than those siliques in the same inflorescence which were self-fertilized (Figures 7). These results confirm that the unfertilized ovaries are mostly, if not exclusively, caused by pollen defects in the *AtrabD2b/2c *mutant.

### *In silico *and GUS Analysis of *AtRabD2b *and *AtRabD2c *Expression

**Figure 8:**
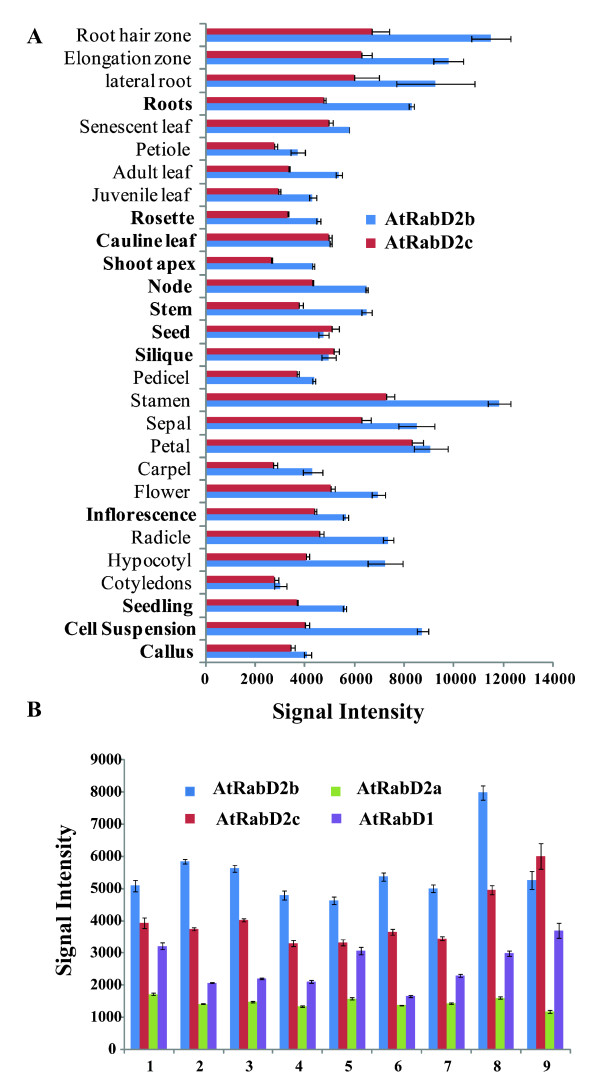
***In silico *expression analysis of *AtRabD2b *and *AtRabD2c***. The spatial and temporal expression profiles of *AtRabD2b *and *AtRabD2c *were analyzed using Genevestigator anatomy (A) and development (B) tools, respectively. Numbers along the X axis represent the developmental stage: 1, germinated seed; 2, seedlings; 3, young rosette; 4, developed rosette; 5, bolting; 6, young flower; 7, developed flower; 8, flowers and siliques; 9, mature siliques.

If AtRabD2b and AtRabD2c are involved in pollen development and pollen tube growth, they are expected to be co-expressed in pollen and pollen tubes. Public microarray data indicates that both AtRabD2b and AtRabD2c are expressed throughout development, including high expression in floral organs and particularly in the stamen (Figure 8; [25,26]).

**Figure 9:**
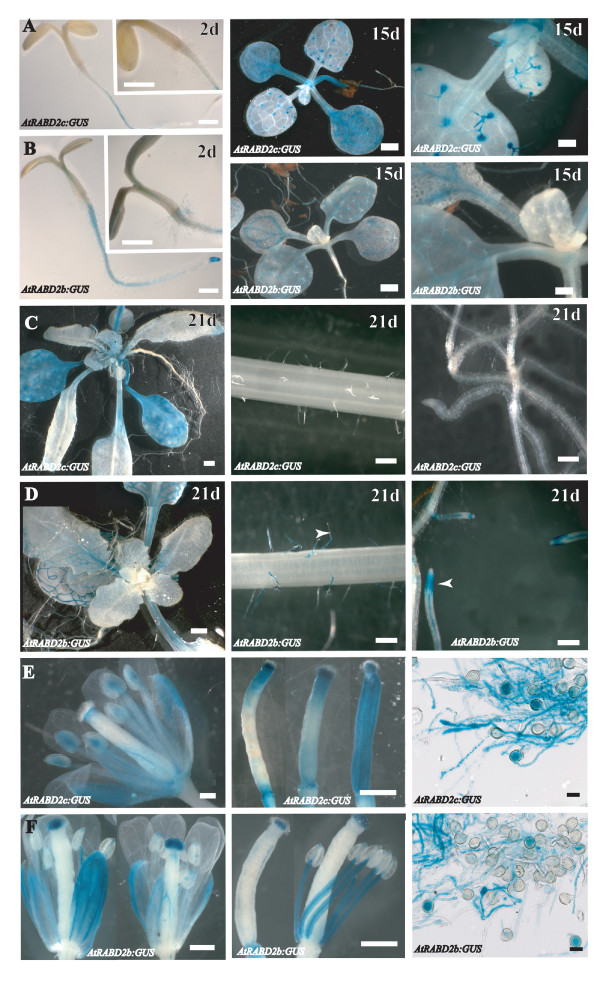
**Temporal and spatial expression pattern of *AtRabD2b *and *AtRabD2c***. Transgenic plants were generated that express the GUS gene driven by a 954bp or 558bp fragment upstream of the *AtRabD2b *or *AtRabD2c *start codon, respectively. GUS activity (blue color) was analyzed in cotyledons, young leaves, (A, B), old leaves, roots (C, D), flowers, pistils and germinated pollen (E, F).

To directly examine the spatial expression pattern of the *AtRabD2b *and *AtRabD2c *genes, transgenic lines containing promoter:GFP/GUS constructs for each gene were analyzed for GUS activity at various stages of development from germination to senescence. As indicated by the *in silico *analyses, both *AtRabD2b *and *AtRabD2c *were expressed widely during development. GUS staining further indicated that in cotyledons, rosette leaves and cauline leaves, AtRabD2b expression was localized predominantly in vascular tissues (Figure 9B), whereas AtRabD2c was expressed ubiquitously in cotyledons and in mature leaves throughout the entire leaf. Interestingly, in emerging leaves, AtRabD2c was only expressed in the trichomes, while AtRabD2b was not expressed in these cells (Figure 9A). In flowers, AtRabD2b was expressed in sepals, stamen and stigma, while AtRabD2c was expressed in sepal, stamen, stigma and style (Figure 9E, F). This dichotomy of expression suggests that AtRabD2b and AtRabD2c may function independently of each other in certain cells. Both genes were expressed in pollen grains and germinating pollen (Figure 9E, F), which is consistent with their role in pollen development and pollen tube growth. They both also showed expression in roots, but AtRabD2b was ubiquitously expressed throughout the root, while AtRabD2c expression was excluded from root hairs and root tips (Figure 9A-D).

### Subcellular localization of AtRabD2b and AtRabD2c

**Figure 10:**
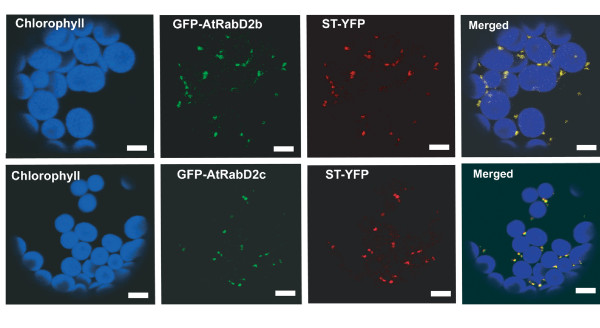
**AtRabD2b and AtRabD2c are both Golgi resident proteins**. Arabidopsis leaf protoplasts were co-transformed with GFP-AtRabD2b or GFP-AtRabD2c and the Golgi marker ST-YFP. GFP, YFP and chlorophyll autofluorescence were detected by confocal microcopy. At least 10 transformed protoplasts were observed for each construct. Upper panel, GFP-AtRabD2b; lower panel, GFP-AtRabD2c. Scale bar = 10 μm.

In mammals, different AtRabD orthologs (Rab1 isoforms) are localized to the ER, ER-Golgi intermediate compartment or Golgi compartments. In plants, AtRabD2a and AtRabD2b are associated with the Golgi apparatus and *trans*-Golgi network [15,22,23,27], and we predicted that AtRabD2c will share this localization. To determine the subcellular localization of AtRabD2c, GFP-AtRabD2b and GFP-AtRabD2c constructs were introduced into Arabidopsis leaf protoplasts and the GFP signal was observed by confocal microscopy. For both constructs, GFP localized to punctuate structures, reminiscent of the Golgi apparatus. To verify the identity of these structures, GFP-AtRabD2b and GFP-AtRabD2c were co-transfected into Arabidopsis leaf protoplasts with the *trans*-Golgi marker ST-YFP [15], and YFP and GFP signals were observed. Confocal results indicated that both AtRabD2b and AtRabD2c primarily colocalized with ST-YFP (Figure 10) and are therefore associated with the Golgi, consistent with a role in Golgi trafficking. Occasionally, AtRabD2b or AtRabD2c-labeled structures were seen that did not contain ST-YFP; these could be the post-Golgi compartments described previously [22,23,27]. Cells expressing a single GFP or YFP fusion demonstrated the absence of cross talk between GFP and YFP signals (Additional file 3, Figure S2).

## Discussion

Rab GTPases are critical players in the transport of materials through the endomembrane system, controlling exocytosis of proteins and cell wall materials, endocytosis of receptors and transporters, and membrane recycling processes. Together with SNARE proteins, they promote specificity of vesicle transport to target compartments, ensuring that vesicles fuse only with their appropriate target and thus maintaining the distinct identity of individual organelles. Two Rab subfamilies, Rab1 (AtRabD orthologs) and Rab2 (AtRabB orthologs), have been reported to function in membrane trafficking between the ER and Golgi in mammalian cells [21,28-34]. The plant Rab1 and Rab2 homologs AtRabD1, AtRabD2a and NtRab2 have also been reported to function in ER to Golgi vesicle transport [15,16]. Here, we demonstrate a distinct physiological role for the Rab1 homologs AtRabD2b and AtRabD2c in pollen development and pollen tube growth.

Using the bioinformatics tool MetaOmGraph (http://www.metnetdb.org) [25,26] to determine the pairwise Pearsons correlation value between the expression patterns of all of the 57 AtRab genes (Additional file 1, Table S1), we found that among the four AtRabDs, only the expression of AtRabD2b and AtRabD2c are highly correlated. From this data, we hypothesized that AtRabD2b and AtRabD2c have partially redundant functions that are not shared by the remaining AtRabD family members. To test our hypothesis, we used T-DNA insertion single and double mutants to confirm that AtRabD2b and AtRabD2c have functional overlap and show that they are both required for normal pollen development and tip growth of pollen tubes. We also showed that they both co-localize with the *trans*-Golgi marker ST-YFP, consistent with their proposed role in Golgi trafficking.

The conclusion that AtRabD2b and AtRabD2c are partially functionally redundant is based on several lines of evidence. First, although single mutant plants containing *AtrabD2b *or *AtrabD2c *mutant alleles are indistinguishable morphologically from their wild-type counterparts, even when grown under a variety of growth conditions, the *AtrabD2b/2c *double mutant has a short-silique phenotype. Second, both *AtrabD2b *and *AtrabD2c *mutant plants produce a small percentage of deformed and collapsed pollen grains, while *AtrabD2b/2c *lines produce a higher percentage of deformed pollen grains, many of which are severely deformed, some lacking nuclei. It is probable that such aberrant pollen would give rise to defects in pollen germination, and indeed, though the germination rate is similar between *AtrabD2b *or *AtrabD2c *single mutants and wild-type plants, about 10% of the pollen grains from *AtrabD2b/2c *double mutant plants are unable to geminate. Furthermore, *AtrabD2b *and *AtrabD2c *mutant pollen tubes do not grow apically as well as do wild-type pollen tubes and tend to have swollen tips and a shorter length; this phenotype is substantially more severe in *AtrabD2b/2c *double mutants. In addition, some pollen tubes from *AtrabD2b/2c *double mutants branch or burst, which is not seen in pollen tubes of wild-type plants or either single mutant. These data also indicate that the loss of function of the *AtRabD2b/2c *genes cannot be compensated for by the AtRabD1 or AtRabD2a genes, suggesting that either some function(s) of the AtRabD2b and AtRabD2c proteins are distinct from those of AtRabD1 or AtRabD2a, or that they are not expressed in the same cell types.

Both AtRabD2b and AtRabD2c co-localize with the Golgi marker ST-YFP upon transient expression in Arabidopsis leaf protoplasts, as was reported also for AtRabD2a (formerly called AtRab1b) [15]. It is therefore possible that AtRabD2b and AtRabD2c function in vesicle trafficking between the ER and Golgi apparatus, as does AtRabD2a [15,22]. Complete disruption of Rab function in ER-to-Golgi trafficking is expected to be lethal, due to loss of plasma membrane, vacuole and cell wall assembly and integrity. However, the *AtrabD2b/2c *double mutant is indistinguishable from wild-type plants, except for shorter siliques due to the pollen and pollen tube defects. There are several possible explanations for this. First, other AtRabs must perform the same function in vegetative tissue. The most likely candidates are AtRabD2a and AtRabD1, which could compensate for the loss of function in the *AtrabD2b/2c *mutant in most cell types [22]. Moreover, other Rab families, such as tobacco RabBs (NtRab2s) have also been shown to be regulators of membrane trafficking between the ER and Golgi apparatus [16]. AtRabBs (AtRab2s) may have the same function, such that they also participate in ER to Golgi vesicle trafficking. The pattern of AtRabB1b RNA accumulation is most highly correlated with that of AtRabD2b (67%) and AtRabD2c (68%) (Additional file 1, Table S1). These genes might compensate in part for the loss of function of *AtrabD2b/2c*.

Second, pollen tubes grow very rapidly compared with many other cell types. Pollen tubes elongate by tip growth, whereby the pollen cytoplasm is confined to the most proximal region of the tube, and growth is restricted to the tube apex [35]. *In vitro*, lily pollen tubes grow at about 150 nm/sec [35] and Arabidopsis pollen tubes at 37 nm/sec [36]; *in vivo*, tobacco pollen can grow at 42 nm/sec [37]. This fast growth is contingent on rapid vesicle trafficking to deliver large amounts of membrane and cell wall components to the apical region of the tubes. This extensive trafficking requirement may preclude the remaining Rabs from completely compensating for loss of AtRabD2b and AtRabD2c.

Third, computational analysis of public microarray data, together with studies of the expression pattern directed by the AtRabD2b and AtRabD2c promoters, indicated that both are widely expressed in most organs and several cell types, with high expression in pollen. Root hairs also showed expression of AtRabD2b, and, like pollen tubes, root hairs elongate by tip growth. However, root hair growth in the *AtrabD2b/2c *double mutant is indistinguishable from that of wild-type plants. This is consistent with the idea that AtRabD2b and AtRabD2c are required for vesicle trafficking in multiple cell types, and that the highest demand for this process may be in pollen and pollen tubes, in order to optimally support the large secretory requirement of these very rapidly elongating cells. In combination, these data indicate that the high expression of AtRabD2b and AtRabD2c in pollen may be important to facilitate membrane trafficking needed for pollen tube growth.

## Conclusions

In summary, we used a T-DNA insertion mutant approach to demonstrate the function of AtRabD2b and AtRabD2c. Our data indicated that both are Golgi residents; they have similar but not identical expression patterns, but are both highly expressed in pollen; they are both involved in tip growth of pollen tubes; and they are at least partially functionally redundant. Future work will focus on elucidating the molecular basis for the pollen phenotype in the *AtrabD2b/2c *double mutant.

## Methods

### Plant Materials and Growth Conditions

Wild-type Arabidopsis (*Arabidopsis thaliana*) ecotype Columbia (Col-0), *AtrabD2c-1*, *AtrabD2b-1 *and *AtrabD2b/2c *(crosses of *AtrabD2b-1 *and *AtrabD2c-1*) mutants in the same genetic background were used. Seeds were sown in Sunshine Soil mix, incubated at 4°C for 2 to 3 days, then grown at 22°C, 70% relative humidity, in a 16-h light/8-h dark photoperiod [26].

### Screening for T-DNA insertion mutants

T-DNA insertion mutants of *AtRabD2b *and *AtRabD2c *(Salk_045030 (*AtrabD2b-1*), Salk_117532 (*AtrabD2b-2*) and Salk_120116 (*AtrabD2b-3*) for AtrabD2b; Salk_054626 (*AtrabD2c-3*) for AtrabD2c) were obtained from ABRC [38]. Homozygous lines for T-DNA insertions were identified by PCR genotyping. For each T-DNA insertion mutant, two sets of PCRs were performed using genomic DNA as a template: one with a gene-specific primer and a T-DNA left border primer LBb1, the second with two gene-specific primers. The PCR products were sequenced to confirm the locations of the T-DNA insertion sites for all of the mutants. The gene specific primers used are listed in Table 2.

**Table 2:** Primers used in this study

LBb1	GCGTGGACCGCTTGCTGCAACT
AtrabD2b-LP1	CCCTTCGTTGGGCTAGTAAAG

AtrabD2b-RP1	TTCAACAACGTCAAACAATGG

AtrabD2c-LP1	GCGCATTACTGAGAGAGAAGAG

AtrabD2c-RP1	TCCCATTCTTGGAAACAAGTG

AtRabD2b-F	ATGAATCCTGAATATGACTAT

AtRabD2b-R	TCAAGAAGAACAACAGCCT

AtRabD2c-F	ATGAATCCTGAATATGACTAT

AtRabD2c-R	TTAAGAGGAGCAGCAGCCT

AtRabD2b-g-F	caccATCGCTTATCCGCTCCGTGTATTTC

AtRabD2b-g-R	TAAAGACCCCTGGTCCTTCAGC

AtRabD2c-g-F	caccCTATCTCACTAAGCTGAAGATAC

AtRabD2c-g-R	GGCAATCTCTCCGGTTTGGTCC

β-Tubulin-F	CGTGGATCACAGCAATACAGAGCC

β-Tubulin-R	CCTCCTGCACTTCCACTTCGTCTTC

### Crossing and screening for double mutant

Single mutant alleles (*AtrabD2b-1 *and *AtrabD2c-1*; Figure 1A) were crossed, the F1 generation of these crosses was allowed to self fertilization and the *AtrabD2b/2c *double mutant was identified from the F2 generation by PCR genotyping.

### Semi-quantitative reverse transcription PCR

Total RNA was extracted from leaves of 20 DAI (days after imbibition) plants using the TRIZOL reagent (Invitrogen). RT-PCR was performed using SuperScript™ III One-Step RT-PCR System (Invitrogen,) as per the manufacturer's manual. The β-tubulin gene, which is highly conserved and constitutively expressed in all eukaryotes, was used as a standard. The primers used are listed in Table 2. The RT-PCR products were sequenced to confirm the correct amplification product.

### *In vitro *pollen germination and growth measurement

Pollen was obtained from flowers collected from Arabidopsis plants (ten plant lines per genotype) 1 to 2 weeks after bolting. Pollen from *AtrabD2b/2c*, *AtrabD2b *and *AtrabD2c *mutants, along with pollen from wild-type plants, was germinated on agar medium containing 18% (w/v) sucrose, 0.01% (w/v) boric acid, 1mM MgSO_4_, 1mM CaCl_2_, 1mM Ca(NO_3_)_2_, and 0.5% (w/v) agar, pH 7.0 [39] overnight at room temperature and examined and photographed under a Zeiss Axioplan II compound microscope equipped with an AxioCam color digital camera. Measurements were performed using SIS Pro software (OSIS, Lakewood, CO) using the bars in the original image. For pollen tube length measurements, 200 pollen tubes were chosen randomly for each genotype, and significance was assessed using Student's *t*-test.

For fluorescence microscopy, the germinated pollen was transferred onto a slide and two drops of aniline blue solution (0.005% aniline blue solution in 0.1 M sodium phosphate, pH 7.0) were added for ten minutes.

To confirm the pollen tube growth defects, 20 open flowers per genotype were cut below the pistil and inserted vertically into germination medium in a 9-cm Petri dish. Plates were sealed and incubated overnight at 22°C at 100% humidity under continuous illumination. The paths of pollen tubes inside the pistils were visualized by fixing whole pistils in 2% glutaraldehyde and 2% paraformaldehyde in 0.1 M sodium cacodylate buffer, pH 7.2, under low vacuum (18 psi Hg) for 2 h at room temperature. Samples were washed three times in the same buffer and stained with Aniline Blue and DAPI. The tissue was then cleared for 24 hours at room temperature with a drop of clearing solution (240 g of chloral hydrate and 30 g of glycerol in 90 ml water). Pollen was examined with a Zeiss Axioplan 2 light microscope (LM) and images were captured with a Zeiss AxioCamHRc digital camera (Carl Zeiss,Inc., Thornwood, NY) using AxioVision 4.3 software. The microscope was equipped with a DAPI filter set comprising an excitation filter (BP 365/12 nm), a beam splitter (395 nm), and an emission filter (LP 397 nm). The objectives used for imaging were a Neofluar 40× oil, an Apochromat 63× oil, and a Neofluar 100× oil.

### Cloning

Promoter::GFP/GUS fusion constructs were made for each gene by cloning the amplified promoter region (intergenic region; 964 bp for AtRabD2b and 558 bp for AtRabD2c) into the binary vector pBGWFS7 (GATEWAY; Invitrogen).

The genomic fragments containing AtRabD2b or AtRabD2c with their respective promoters for complementation of the mutant phenotype were amplified using AtRabD2b-g-F and R or AtRabD2c-g-F and R primers (Table 2). Products were cloned into the pENTR/D vector (Invitrogen), and then were transferred into the pMDC123 binary vector for plant transformation.

### Plant transformation and selection

Arabidopsis plants were transformed using *Agrobacterium tumefaciens *by the floral dip method [40] and selected for Basta resistance conferred by the T-DNA.

### Transcriptomic analysis

MetaOmGraph (MOG; http://www.metnetdb.org) [25] was used to analyze expression patterns of AtRabD1, AtRabD2a, AtRabD2b and AtRabD2c and derive the correlation between them.

### GUS assay

Transgenic T2 seedlings were germinated in soil and harvested at various stages of development. Plants or organs were stained at room temperature overnight as described [41], then destained in 70% (v/v) ethanol. For each construct, at least 7 independently transformed lines, 7 plants for each stage, were harvested for GUS screening.

### Transient expression in protoplasts

Transient gene expression in Arabidopsis mesophyll protoplasts was carried out as described previously [42]. In brief, Arabidopsis protoplasts were isolated from the leaves of 3-4 week old plants. Leaf strips were digested in a buffer containing cellulose R-10 and macerozyme R-10. After adding 30 μg of plasmid DNA, an equal volume of protoplasts was mixed with PEG buffer (40% (w/v) PEG4000, 25% (v/v) 0.8M mannitol, 10% 1M CaCl_2_) then incubated at room temperature for 25 min. After gentle washing, the protoplasts were kept in the dark at room temperature overnight and then viewed by confocal laser scanning microscopy as described below.

### Confocal laser scanning microscopy

Colocalization of GFP-RabD2b and GFP-RabD2c with ST-YFP was performed using a Leica TCS SP10 confocal microscope, which allows flexible selection of emission bandwidths to minimize bleed-through. Transformed cells were excited with a 488 nm laser (power 20%) and 514 nm laser (50% power), and GFP and YFP signals were collected using 495-510 nm and 560-640 nm bandwidths, respectively. Non-transformed cells and cells expressing a single GFP or YFP fusion were used as controls to confirm the absence of cross talk between GFP, YFP and autofluorescence signals.

### Scanning electron microscopy

Pollen that had been germinated *in vitro *was placed in 2% glutaraldehyde and 2% paraformaldehyde in 0.1 M sodium cacodylate buffer, pH 7.2, under low vacuum (18 psi Hg) for 5 h at room temperature. Samples were washed three times in the same buffer, postfixed in 1% osmium tetroxide in the same buffer for 2 h and washed two times in the same buffer, followed by deionized water. Samples were dehydrated through a graded ethanol series (50, 70, 85, 95, and 100%; 30 min per step), followed by two changes of ultrapure 100% ethanol, all 30 min per step. Fresh pollen was also examined without fixing. Fixed samples were critical point-dried in a DCP-1 Denton critical-point-drying apparatus (http://www.dentonvacuum.com) using liquid carbon dioxide, and mounted on aluminum stubs with double-sided sticky pads and silver cement.

Samples were then sputter-coated with 15 nm gold (20%) and palladium (80%) in a Denton Vacuum LLC Desk II Cold Sputter Unit (http://www.dentonvacuum.com), and viewed with a JEOL 5800LV SEM (http://www.jeol.com) at 10 kV. Alternatively, released fresh pollen grains were directly mounted on stubs and sputter-coated with gold particles before SEM analysis. All digitally collected images including the LM and SEM images were processed in Adobe PhotoShop 7.0 and made into plates using Adobe Illustrator 10. Over 20 samples from each plant line were used for SEM or LM analysis.

## Authors' contributions

JP carried out the experimental analyses described and drafted the manuscript. HI helped with the microscopy and figures. ESW conceived of the study, participated in its design and analysis of the data and helped to draft the manuscript. DCB participated in the design of the study, analysis of the data and helped to draft the manuscript. All authors read and approved the final manuscript.

## Supplementary Material

Additional file 1 **Table S1. Expression pattern of AtRab genes**. Pearson correlation between expression patterns of AtRab genes determined using MetaOmGraph (Excel file). Click here for file

Additional file 2 **Figure S1. Seed number per silique in wild-type and mutant plants**. Seed number was counted for 15 siliques of 5 individual plants for the indicated genotypes. Error bars indicate standard deviation (pdf file). Click here for file

Additional file 3 **Figure S2. Controls for confocal microscopy**. Arabidopsis leaf protoplasts were transformed with either GFP-AtRabD2b or ST-YFP and imaged in the green, yellow and red channels as shown in Figure 10. No cross-talk between channels could be seen using these settings. Upper panel, GFP-AtRabD2b; lower panel, ST-YFP. Scale bar = 10 μm (pdf file). Click here for file
