# Supplementary figures and images for: 5AtRabD2b and AtRabD2c have overlapping functions in pollen development and pollen tube growth
Source: BMC Plant Biol. 2011 Jan 26;11:25. doi: 10.1186/1471-2229-11-25 (PMC3040128; doi:10.1186/1471-2229-11-25)

Seeds per silique

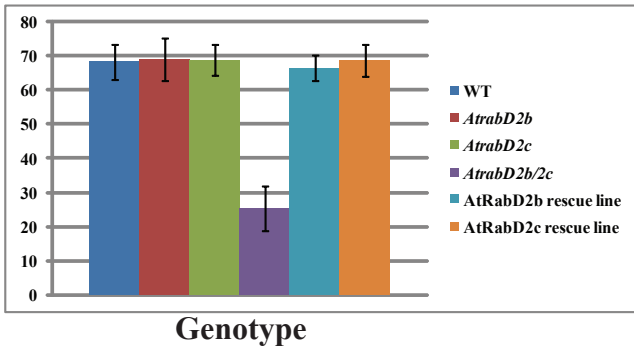

Supplement: Additional file 2 — Figure S1. Seed number per silique in wild-type and mutant plants. Seed number was counted for 15 siliques of 5 individual plants for the indicated genotypes. Error bars indicate standard deviation (pdf file). [file 1471-2229-11-25-S2.PDF]

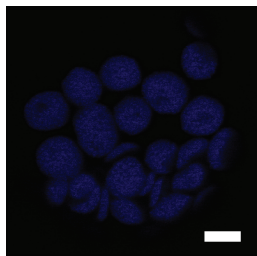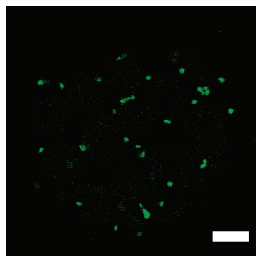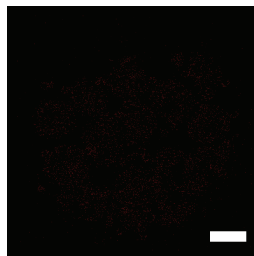

**GFP-AtRabD2b**

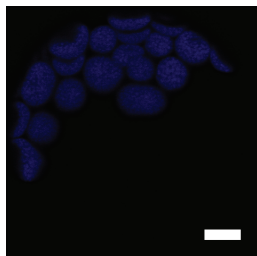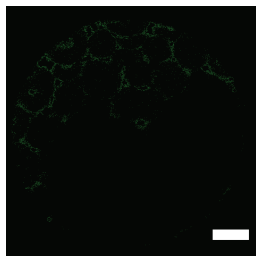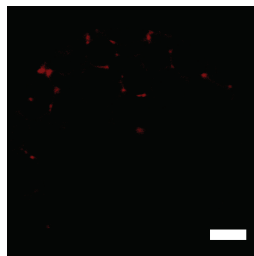

**ST-YFP**

**chlorophyll**

**green channel**

**yellow channel**

Supplement: Additional file 3 — Figure S2. Controls for confocal microscopy. Arabidopsis leaf protoplasts were transformed with either GFP-AtRabD2b or ST-YFP and imaged in the green, yellow and red channels as shown in Figure 10. No cross-talk between channels could be seen using these settings. Upper panel, GFP-AtRabD2b; lower panel, ST-YFP. Scale bar = 10 μm (pdf file). [file 1471-2229-11-25-S3.PDF]
